# A Hereditary Enteropathy Caused by Mutations in the *SLCO2A1* Gene, Encoding a Prostaglandin Transporter

**DOI:** 10.1371/journal.pgen.1005581

**Published:** 2015-11-05

**Authors:** Junji Umeno, Tadakazu Hisamatsu, Motohiro Esaki, Atsushi Hirano, Naoya Kubokura, Kouichi Asano, Shuji Kochi, Shunichi Yanai, Yuta Fuyuno, Katsuyoshi Shimamura, Naoki Hosoe, Haruhiko Ogata, Takashi Watanabe, Kunihiko Aoyagi, Hidehisa Ooi, Kenji Watanabe, Shigeyoshi Yasukawa, Fumihito Hirai, Toshiyuki Matsui, Mitsuo Iida, Tsuneyoshi Yao, Toshifumi Hibi, Kenjiro Kosaki, Takanori Kanai, Takanari Kitazono, Takayuki Matsumoto

**Affiliations:** 1 Department of Medicine and Clinical Science, Graduate School of Medical Sciences, Kyushu University, Fukuoka, Japan; 2 Division of Gastroenterology and Hepatology, Department of Internal Medicine, Keio University School of Medicine, Tokyo, Japan; 3 The Third Department of Internal Medicine, Kyorin University School of Medicine, Mitaka, Japan; 4 Department of Gastroenterology, Matsuyama Red Cross Hospital, Matsuyama, Japan; 5 Center for Diagnostic and Therapeutic Endoscopy, Keio University School of Medicine, Tokyo, Japan; 6 Department of Gastroenterology, Fukuoka University School of Medicine, Fukuoka, Japan; 7 Division of Gastroenterology, Imamura Hospital, Kagoshima, Japan; 8 Department of Gastroenterology, Osaka City University Graduate School of Medicine, Osaka, Japan; 9 Department of Gastroenterology, Fukuoka University Chikushi Hospital, Chikushino, Japan; 10 Kyushu Central Hospital, Fukuoka, Japan; 11 Sada Hospital, Fukuoka, Japan; 12 Center for Advanced IBD Research and Treatment, Kitasato University Kitasato Institute Hospital, Tokyo, Japan; 13 Center for Medical Genetics, Keio University School of Medicine, Tokyo, Japan; 14 Division of Gastroenterology, Department of Internal Medicine, Faculty of Medicine, Iwate Medical University, Morioka, Japan; The University of North Carolina at Chapel Hill, UNITED STATES

## Abstract

Previously, we proposed a rare autosomal recessive inherited enteropathy characterized by persistent blood and protein loss from the small intestine as chronic nonspecific multiple ulcers of the small intestine (CNSU). By whole-exome sequencing in five Japanese patients with CNSU and one unaffected individual, we found four candidate mutations in the *SLCO2A1* gene, encoding a prostaglandin transporter. The pathogenicity of the mutations was supported by segregation analysis and genotyping data in controls. By Sanger sequencing of the coding regions, 11 of 12 other CNSU patients and 2 of 603 patients with a diagnosis of Crohn’s disease were found to have homozygous or compound heterozygous *SLCO2A1* mutations. In total, we identified recessive *SLCO2A1* mutations located at seven sites. Using RT-PCR, we demonstrated that the identified splice-site mutations altered the RNA splicing, and introduced a premature stop codon. Tracer prostaglandin E2 uptake analysis showed that the mutant SLCO2A1 protein for each mutation exhibited impaired prostaglandin transport. Immunohistochemistry and immunofluorescence analyses revealed that SLCO2A1 protein was expressed on the cellular membrane of vascular endothelial cells in the small intestinal mucosa in control subjects, but was not detected in affected individuals. These findings indicate that loss-of-function mutations in the *SLCO2A1* gene encoding a prostaglandin transporter cause the hereditary enteropathy CNSU. We suggest a more appropriate nomenclature of “chronic enteropathy associated with *SLCO2A1* gene” (CEAS).

## Author Summary

Advanced diagnostic innovations such as capsule endoscopy and balloon endoscopy have provided better understanding of endoscopic findings of small bowel diseases. However, it remains difficult to diagnose small intestinal diseases such as Crohn’s disease, intestinal tuberculosis, and nonsteroidal anti-inflammatory drug-induced enteropathy by the endoscopic findings alone. We previously reported a rare autosomal recessive inherited enteropathy characterized by persistent blood and protein loss from the small intestine. This enteropathy has an intractable clinical course with ineffectiveness of immunosuppressive treatment. In this study, we identified recessive mutations in the *SLCO2A1* gene, encoding a prostaglandin transporter, as causative variants of this disorder by exome sequencing of four families, and showed that this disease is distinct from Crohn’s disease. We also showed that the mutations found in the patients caused functional impairment of prostaglandin E2 uptake within cells. The present findings suggest that genetic analysis together with detailed clinical information is invaluable for diagnosis of the disease, and that there may be a concept of enteropathy referred to as “prostaglandin-associated enteropathy”, irrespective of ethnic background.

## Introduction

The use of capsule endoscopy and balloon endoscopy has provided a better understanding of the features of small bowel ulcers among various gastrointestinal disorders, such as Crohn’s disease (CD), intestinal tuberculosis, and nonsteroidal anti-inflammatory drug (NSAID)–induced enteropathy [1,2]. Previously, we proposed a rare clinicopathologic entity characterized by multiple small intestinal ulcers of nonspecific histology and chronic persistent gastrointestinal bleeding as chronic nonspecific multiple ulcers of the small intestine (CNSU) [3,4]. The macroscopic findings of CNSU are characterized by multiple thin ulcers in a linear or circumferential configuration and concentric stenosis, and apparently mimic those of NSAID-induced enteropathy [4–7]. CNSU predominantly occurs in females and the symptoms, such as general fatigue, edema, and abdominal pain, appear during adolescence. The clinical course of the disease is chronic and intractable with reduced effects of immunosuppressive treatment including prednisolone and azathioprine.

Although CNSU predominantly occurs in females, it also appears to be an autosomal recessive inherited disorder because of frequent parental consanguinity [8]. To identify the causative gene for this disorder, we performed whole-exome sequencing and identified recessive mutations in the *SLCO2A1* gene, encoding a prostaglandin transporter, as causative variants. Furthermore, we replicated our findings in other patients with CNSU and established a genetic cause for this inherited disease.

## Results

### Whole-exome sequencing

**Fig 1:**
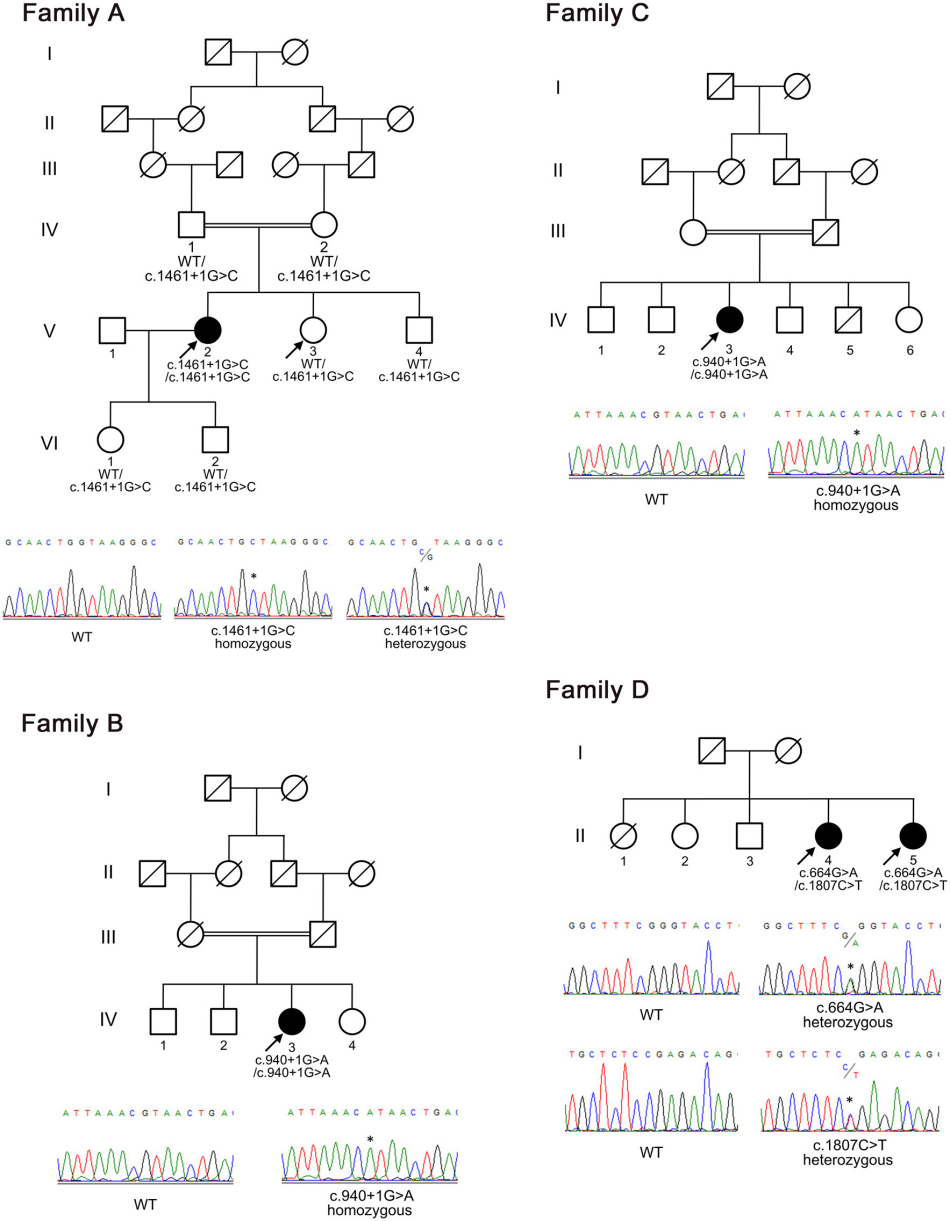
Pedigrees of the families with chronic nonspecific multiple ulcers of the small intestine. The segregation of the *SLCO2A1* mutations c.1461+1G>C (splice site, family A), c.940+1G>A (splice site, families B and C), c.664G>A (G222R, family D), and c.1807C>T (R603X, family D) is indicated. Squares represent male family members, circles represent female family members, black symbols represent clinically affected family members, and slashes represent deceased family members. Arrows indicate individuals whose DNA was analyzed by whole-exome sequencing. WT denotes wild-type.

**Fig 2:**
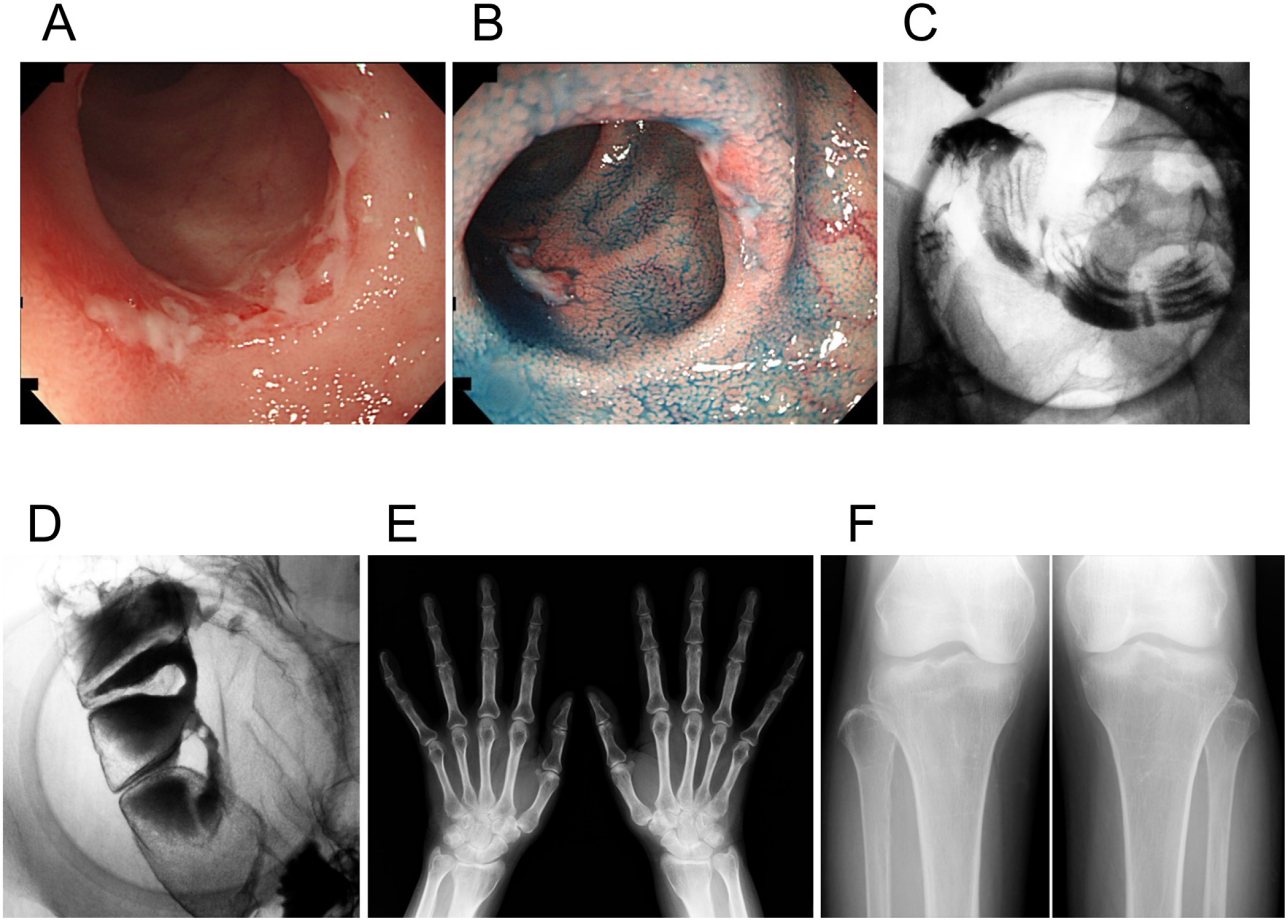
Clinical images of an individual with chronic nonspecific multiple ulcers of the small intestine (patient A-V–2). (A, B) Retrograde ileoscopy shows active circular and oblique multiple ulcers with mucous exudates in the ileum. (C, D) A barium follow-through examination with compression shows multiple circular barium flecks (C), eccentric deformities, and strictures (D) in the ileum. (E, F) Radiographs of the hands and tibiofibulae show no obvious abnormalities such as cortical thickening of the metacarpals and periosteal hyperostosis.

We performed whole-exome sequencing in five affected females with CNSU (A-V–2, B-IV–3, C-IV–3, D-II–4, and D-II–5) and one unaffected individual (A-V–3) (Figs 1 and 2). Parental consanguinity was noted in families A, B, and C. The average depth of sequence coverage in the whole-exome sequencing data was 68.9× (S1 Table). We identified a total of 368,403 variants, of which 20,271 were non-synonymous or splice-site mutations.

By filtering the data with dbSNP135, we found 2,406 variants located in 1,578 genes. Based on the parental consanguinity of the patients, we focused on the shared genes with homozygous variants among three affected individuals (A-V–2, B-IV–3, and C-IV–3) and found nine candidate genes, *PCSK9*, *ASPM*, *DAG1*, *SLCO2A1*, *MCPH1*, *EFEMP2*, *DDHD1*, *PKD1L3*, and *SYNGR1*. After consideration of the results for the unaffected individual (A-V–3) and another two sibling patients (D-II–4 and D-II–5), only *SLCO2A1* remained as a candidate gene. The three patients with parental consanguinity (A-V–2, B-IV–3, and C-IV–3) had a homozygous splice-site mutation in the *SLCO2A1* gene, c.1461+1G>C or c.940+1G>A (Fig 1 and Table 1). The two sibling patients had compound heterozygous mutations, c.664G>A (p.Gly222Arg) and c.1807C>T (p.Arg603X). All four mutations were predicted to be loss-of-function or damaging mutations by SIFT and PolyPhen–2.

**Table 1:** Clinical features and *SLCO2A1* mutations in Japanese patients with chronic nonspecific multiple ulcers of the small intestine.

Patients	Sex	Consanguinity (degrees)	Family History	*SLCO2A1* Mutation		Age (yr)		Presenting Symptoms	Disease Site	Laboratory Data at Diagnosis			Surgery
						Onset	Diagnosis			Hemoglobin (g/dl)	Serum Protein (g/dl)	CRP (mg/dl)	
1 (A-V–2)	F	Yes (5)	No	SS/SS	c.1461+1G>C/ c.1461+1G>C	17	43	Anemia	I	9.6	4.6	0.5	+
2 (B-IV–3)	F	Yes (3)	No	SS/SS	c.940+1G>A/ c.940+1G>A	37	38	Anemia	S, I	9.5	6.7	0.5	+
3 (C-IV–3)	F	Yes (3)	No	SS/SS	c.940+1G>A/ c.940+1G>A	11	39	Anemia, abdominal pain	I	NA	NA	NA	+
4 (D-II–4)	F	No	Yes	NS/NS	Gly222Arg/ Arg603X	53	55	Anemia	S, I	9.7	5.2	0.1	-
5 (D-II–5)	F	No	Yes	NS/NS	Gly222Arg/ Arg603X	12	22	Anemia, abdominal pain	S, D, I	9.7	5.8	0.3	+
6	F	No	Yes	SS/SS	c.940+1G>A/ c.940+1G>A	12	51	Anemia	D, I	4.8	5.3	0.0	+
7	F	No	Yes	SS/SS	c.940+1G>A/ c.940+1G>A	16	41	Anemia	D, I	10.7	5.8	0.9	+
8	F	Yes (3)	No	SS/SS	c.940+1G>A/ c.940+1G>A	13	29	Anemia	D, I	8.4	5.0	0.2	+
9	F	Yes (3)	No	NS/NS	Val458Phe/ Val458Phe	40	66	Anemia, hypoproteinemia	I	9.5	4.4	0.6	+
10	F	No	No	NS/NS	Glu141X/ Arg603X	50	59	Anemia, abdominal pain	I	8.5	6.3	0.1	-
11	M	No	No	SS/SS	c.940+1G>A/ c.940+1G>A	20	41	Anemia, hypoproteinemia	D, J, I	11.0	4.8	1.6	-
12	M	No	No	NS/NS	Gly222Arg/ Gly222Arg	15	63	Anemia, hypoproteinemia	J, I	8.1	5.7	0.4	+
13	F	Yes (3)	No	SS/SS	c.940+1G>A/ c.940+1G>A	51	51	Anemia, abdominal pain	S, I	11.2	6.6	0.1	+
14	F	Yes (3)	Yes	SS/SS	c.940+1G>A/ c.940+1G>A	7	7	Anemia, abdominal pain	S, D, J, I	11.1	5.8	0.1	+
15	F	No	No	SS/NS	c.940+1G>A/ Arg603X	18	23	Anemia, abdominal pain	D, I	7.8	3.8	0.0	+
16	M	Yes (NA)	No	SS/SS	c.940+1G>A/ c.940+1G>A	12	31	Anemia, edema	D, I	7.4	8.2	0.1	+
17	M	No	No	SS/NS	c.940+1G>A/ Gly183Arg	1	-	Anemia, edema	J, I	2.3	5.1	0.4	+
18	F	No	No	SS/NS	c.940+1G>A/ Glu141X	52	-	Anemia, edema	J, I	9.5	5.2	0.1	-

Whole-exome sequencing was performed on patients 1–5. Patients 6–16 were screened by Sanger sequencing to validate the results of whole-exome sequencing. Patients 17 and 18 were initially diagnosed as Crohn’s disease. NS, non-synonymous mutation; SS, splice-site mutation; S, stomach; D, duodenum; J, jejunum; I, ileum; NA, not available.

### Confirmation of the identified mutations

The four identified *SLCO2A1* mutations were confirmed to be present in five affected individuals (A-V–2, B-IV–3, C-IV–3, D-II–4, and D-II–5) by Sanger sequencing (Fig 1). Segregation analysis of patient A-V–2 revealed that her unaffected parents, sister, brother, daughter, and son carried the heterozygous c.1461+1G>C mutation (Fig 1A).

To compensate for bias in our analysis, such as the possibility of ethnic-specific variants, we genotyped the four candidate variants in 747 unaffected Japanese subjects from our previous genome-wide association study [9]. All mutations except for the c.940+1G>A mutation were absent in controls (Table 2). The c.940+1G>A mutation was found in the heterozygous state in 3 of 747 controls, showing a similar allele frequency of 0.0022 to the public exome database for the Japanese population (HGVD database).

**Table 2:** *SLCO2A1* mutations in individuals with chronic nonspecific multiple ulcers of the small intestine.

No.	Genomic Position chr3 (hg19)	Exon	Nucleotide Change	Predicted Effect*		Mutant Allele Frequency			
						CNSU^†^ (*n* = 16)	Control (*n* = 747)	HGVD	CD^†^ (*n* = 603)
1	133,674,014	4	c.421G>T^‡^	E141X	-	1/32		1/858	1/1194
2	133,673,888	4	c.547G>A^§^	G183R	Deleterious	0/32		0	1/1194
3	133,672,567	5	c.664G>A	G222R	Deleterious	4/32	0	1/858	0
4	133,667,736	7	c.940+1G>A	Splice	-	19/32	3/1494	3/1330	11/1206
5	133,664,028	10	c.1372G>T	V458F	Deleterious	2/32		0	0
6	133,663,938	10	c.1461+1G>C	Splice	-	2/32	0	0	0
7	133,654,625	13	c.1807C>T	R603X	-	4/32	0	0	0

*Mutation pathogenicity according to SIFT, PolyPhen–2, and PROVEAN.

^†^Initial diagnosis.

^‡^The variant differs from rs148547180 (chr3:133674014).

^§^The 547G>A mutation was identified by Sanger sequencing in a genetic screening of Crohn’s disease patients.

CNSU, chronic nonspecific multiple ulcers of the small intestine; HGVD, Human Genetic Variation Database for the Japanese population; CD, Crohn’s disease.

### Sequencing of other CNSU patients

Subsequently, we screened all 14 coding exons and intron-exon boundaries using Sanger sequencing in 12 other CNSU patients, and identified two novel mutations, c.421G>T (p.Glu141X) and c.1372G>T (p.Val458Phe) (Table 1). Eleven of the 12 patients were found to have homozygous (nine patients) or compound heterozygous (two patients) *SLCO2A1* mutations that were rare and predicted to be deleterious by SIFT, PolyPhen–2, and PROVEAN (Table 1). The remaining patient (66-year-old female), who did not have an *SLCO2A1* mutation, was diagnosed as CNSU because of multiple ulcerations in the duodenum and jejunum. Although anti-tumor necrosis factor-α antibody therapy was ineffective, clinical improvement was achieved by enteral nutrition.

### Genetic screening of the *SLCO2A1* gene in CD patients

Because CNSU can be misdiagnosed as CD in some cases, we searched for the six identified mutations of *SLCO2A1* in CD patients to identify concealed CNSU patients. Among 603 patients previously diagnosed as CD [10], two individuals (patients 17 and 18 in Table 1) were found to carry a pair of compound heterozygous *SLCO2A1* mutations, c.940+1G>A/c.547G>A and c.940+1G>A/c.421G>T, respectively. The c.547G>A mutation (p.Gly183Arg), was a novel mutation at a highly conserved site and predicted to be deleterious by *in silico* analysis. The clinical information for the two individuals was reviewed retrospectively, and the diagnosis of CNSU was confirmed. In total, we identified seven deleterious *SLCO2A1* mutations in 18 patients (Table 2).

### Clinical features of genetically confirmed CNSU patients

In total, we found 18 patients with CNSU confirmed by genetic analysis (Table 1); 14 of them were female. In all patients, the ulcers occurred in the ileum (Fig 2A–2D). The stomach and duodenum were affected in five (27.8%) and eight (44.4%) patients, respectively.

Because mutations in the *SLCO2A1* gene, encoding a prostaglandin transporter, have been reported to be the pathogenic cause of primary hypertrophic osteoarthropathy (PHO; OMIM 614441) [11–13], we investigated whether CNSU patients had clinical manifestations of PHO. Although no patients had any clinical manifestations of PHO requiring treatment, mild digital clubbing or periostosis was present in seven of 18 patients (S2 Table). Moreover, three male patients (patients 12, 16, and 17) fulfilled the major clinical criteria for PHO, having digital clubbing, periostosis, and pachydermia. There were no female patients who fulfilled the major clinical criteria (Fig 2E and 2F).

Among the identified *SLCO2A1* mutations, a splice-site mutation of intron 7 (c.940+1G>A) was the most frequent, and nine of the 18 patients were homozygous for this mutation. There were no obvious correlations between the types of mutations and the clinical phenotypes.

Because the *SLCO2A1* gene encodes a prostaglandin transporter that mediates the uptake and clearance of prostaglandins, the urinary levels of prostaglandin E metabolite (PGE-M) were measured. The urinary PGE-M levels in CNSU patients were significantly higher than those in unaffected individuals (*p* = 0.00013; S1 Fig).

### mRNA analysis of *SLCO2A1*

**Fig 3:**
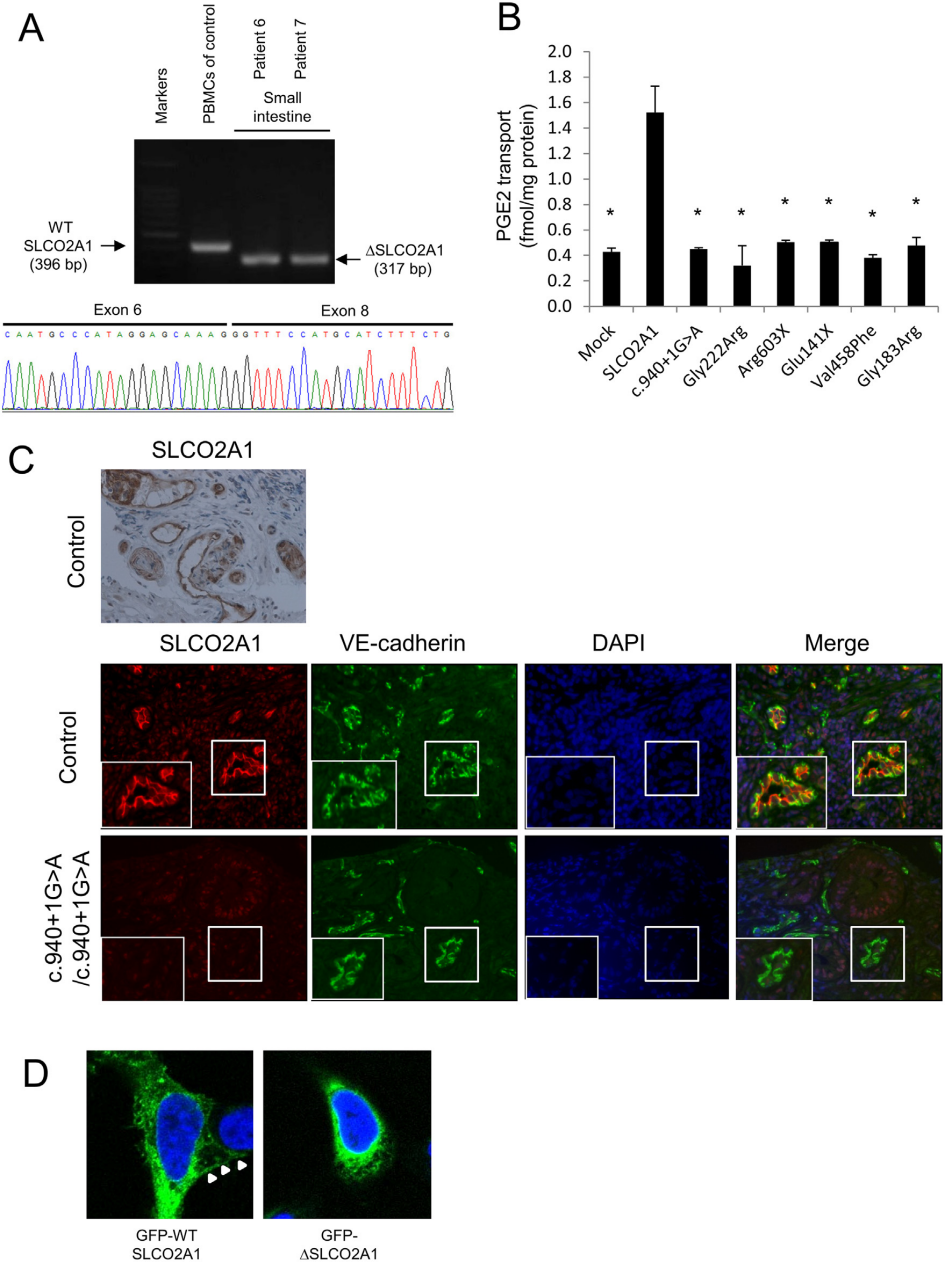
Genetic and functional analyses of *SLCO2A1* gene mutations. (A) RT-PCR and sequencing analysis of *SLCO2A1* mRNA with homozygous c.940+1G>A mutation. A splicing mutation form (deletion of the whole exon 7) of *SLCO2A1* mRNA was expressed in the biopsy specimen from the affected siblings with the homozygous c.940+1G>A mutation (patients 6 and 7). PBMCs denotes peripheral blood mononuclear cells. (B) The mutant SLCO2A1 protein shows loss-of-function for PGE2 transport. Data represent means ± SD (*n* = 3). Statistical analyses were performed using a repeated-measures Dunnett’s multiple-comparison test. *p < 0.0001. (C) Immunohistochemical and immunofluorescence staining using antibodies against SLCO2A1 and VE-cadherin reveals that SLCO2A1 is expressed on the cellular membrane of vascular endothelial cells in the small intestinal mucosa in the control subject, but is not detected in the affected individuals. (D) The truncated form of the SLCO2A1 protein shows altered intracellular localization. Expression vectors for GFP-SLCO2A1 and GFP-ΔSLCO2A1 fusion proteins were transfected into HEK293 cells. GFP-SLCO2A1 protein is localized on the cellular membrane (arrows), while GFP-ΔSLCO2A1 accumulates in the cytosol.

Using RT-PCR, we demonstrated that splicing of the *SLCO2A1* mRNA, derived from biopsy specimens of the small intestine, was altered in affected siblings with the homozygous c.940+1G>A mutation (patients 6 and 7) compared with a control individual (Fig 3A). Sequencing of the RT-PCR products revealed deletion of the whole exon 7 of *SLCO2A1*, leading to a frameshift at amino acid position 288 and introduction of a premature stop codon after six amino acid residues (p.R288Gfs*7). Sequencing of the RT-PCR products of the transcripts in peripheral blood mononuclear cells from patient A-V–2 revealed that the homozygous c.1461+1G>C mutation led to a 23-bp frameshift insertion into intron 10, resulting in a premature stop codon (p.I488Lfs*11) (S2 Fig).

### Functional studies of SLCO2A1 protein

For functional analysis of the intact and truncated SLCO2A1 proteins, we investigated the ^3^H-labeled prostaglandin E2 (PGE2) transport ability in HEK293 cells transfected with intact SLCO2A1 and mutant SLCO2A1 proteins for each identified mutation (c.940+1G>A, p.Gly222Arg, p.Arg603X, p.Glu141X, p.Val458Phe, and p.Gly183Arg). HEK293 cells transfected with intact SLCO2A1 showed the ability for PGE2 transport. In contrast, HEK293 cells transfected with the mutant SLCO2A1 proteins were unable to uptake ^3^H-labeled PGE (*p* < 0.0001; Fig 3B). These findings demonstrated that the mutant SLCO2A1 proteins identified in patients lost their functional ability as a PGE transporter.

In control sections of normal small intestinal mucosa, SLCO2A1 was expressed on the cellular membrane of vascular endothelial cells in the small intestine, as evaluated by immunohistochemistry and immunofluorescence staining with a specific anti-SLCO2A1 antibody recognizing the fifth extracellular domain coded by exons 9–11 of the *SLCO2A1* gene (Fig 3C). We then analyzed the expression of SLCO2A1 in the small intestine of affected individuals with the homozygous c.940+1G>A mutation (patients 6 and 7) by immunofluorescence staining. However, the immunofluorescence staining did not detect any SLCO2A1 protein in the vascular endothelial cells of the patients (Fig 3C). These results indicated that the entire SLCO2A1 protein was unexpressed in affected individuals with the homozygous c.940+1G>A mutation, consistent with the results of the mRNA transcript sequencing.

To investigate the subcellular localization of SLCO2A1 and the truncated SLCO2A1 protein (ΔSLCO2A1) corresponding to the c.940+1G>A mutation, we constructed expression vectors for GFP-SLCO2A1 and GFP-ΔSLCO2A1 fusion proteins and transfected them into HEK293 cells. GFP-SLCO2A1 was localized on the cellular membrane (Fig 3D, arrows) as well as in the cytoplasm of transfected HEK293 cells, while GFP-ΔSLCO2A1 did not accumulate on the cellular membrane (Fig 3D).

## Discussion

In this study, we performed whole-exome sequencing in five Japanese patients with CNSU and one unaffected individual, and identified the *SLCO2A1* gene as the candidate for this disorder. We further confirmed that *SLCO2A1* gene mutations were involved in the pathogenesis of CNSU by genotyping of control subjects and other CNSU patients. Moreover, a genetic analysis of 603 patients previously diagnosed as CD revealed that two CNSU patients had been included in this disease group. In total, we identified seven different mutations in the *SLCO2A1* gene, comprising two splicing-site mutations, two truncating mutations, and three missense mutations, as the causative gene defects for CNSU. Therefore, we propose a more appropriate nomenclature, “chronic enteropathy associated with *SLCO2A1* gene” (CEAS), for this disease.

The *SLCO2A1* gene encodes a prostaglandin transporter that may be involved in mediating the uptake and clearance of prostaglandins in numerous tissues [14–16]. This gene has already been reported as a causative gene for a subtype of PHO [11]. In fact, three of the seven identified mutations, c.664G>A, c.940+1G>A, and c.1807C>T, have also been reported as causative mutations for PHO [11–13,17,18]. We found that three male patients with CEAS had all of the major clinical features of PHO, such as digital clubbing, periostosis, and pachydermia. Moreover, either digital clubbing or periostosis was present in seven of 18 patients. These findings indicate that CEAS and PHO share a causative gene and that their clinical features are profoundly influenced by other modifiers. Taken together with the facts that that CEAS predominantly occurs in females and PHO predominantly occurs in males [8,17], a sex-influenced gene or hormone may be the main disease modifier. Zhang et al. [17] reported that two female family members of a PHO patient had no clinical features of PHO, despite having a homozygous *SLCO2A1* mutation. Moreover, it is interesting to note that these two siblings both had anemia and hypoalbuminemia, suggesting that they had CEAS.

PHO is also known to be caused by mutations of *HPGD*, encoding 15-hydroxyprostaglandin dehydrogenase (15-PGDH), as well as *SLCO2A1* [19]. The transmembrane prostaglandin transporter encoded by the *SLCO2A1* gene delivers prostaglandins to cytoplasmic 15-PGDH, resulting in their degradation [14,20]. Because 15-PGDH is the main enzyme for prostaglandin degradation, systemic PGE2 levels are increased in patients with *HPGD* deficiency. Consistent with the findings in our present investigation, Zhang et al. [11] reported that the urinary levels of PGE2 and PGE-M in SLCO2A1-deficient individuals with PHO are significantly higher than those in controls. In fact, the clinical features of PHO were assumed to be caused by excessive PGE2. Meanwhile, although elevated levels of PGE2 in gastrointestinal tissues are commonly known to protect against mucosal inflammation via the prostaglandin receptor EP3/EP4 [21–23], multiple intestinal ulcers occur in CEAS. This discrepancy and the pathogenesis of intestinal ulcers need to be clarified in future studies.

Although CEAS is presumed to be unaccompanied by immunological inflammation in its pathogenesis, a portion of CEAS patients can be misdiagnosed as CD because of the shared common clinical features, such as multiple small intestinal ulcers, anemia, and hypoalbuminemia. In this study, two of 603 patients previously diagnosed as CD were found to be affected with CEAS by genetic analysis. Because corticosteroid and anti-tumor necrosis factor-α antibody therapies are ineffective for CEAS, recognition and precise diagnosis of CEAS are critical to avoid unnecessary therapies. The findings of our investigation lead us to conclude that genetic analysis in addition to detailed clinical information including digital clubbing, blood tests, and gastrointestinal examinations are invaluable for distinguishing CEAS from CD.

Cases of a similar enteropathy referred to as cryptogenic multifocal ulcerous stenosing enteritis (CMUSE) have been reported in Western populations [24–26]. This enteropathy has been shown to be an autosomal recessive inherited disease caused by mutations in the *PLA2G4A* gene [27]. CEAS and CMUSE share common clinicopathologic features with respect to age of onset, chronic and recurrent clinical course, and nonspecific stenosing small intestinal ulcers [4,25]. However, the sex predominance, response to steroid therapy, and lesion sites are obviously different between the two conditions. The *PLA2G4A* gene encodes cytoplasmic phospholipase A2-α (cPLA2α), which catalyzes the release of arachidonic acid from membrane phospholipids. CMUSE patients with compound heterozygous mutations of *PLA2G4A* have been reported to show globally decreased production of eicosanoids such as PGE2 and thromboxane A2, resulting in multiple ulcers of the small intestine and platelet dysfunction [27,28]. Because impaired prostaglandin use underlies CEAS, CMUSE, and NSAID-induced enteropathy, we propose a new entity of gastrointestinal disorders, namely “prostaglandin-associated enteropathy”.

In conclusion, we have identified loss-of-function mutations in the *SLCO2A1* gene as the cause of CEAS. The present findings clearly indicate that CEAS is a genetically distinct entity independent of other gastrointestinal disorders including CD, NSAID-induced enteropathy, and CMUSE. Further studies are needed to elucidate the pathogenesis of CEAS and identify new therapeutic molecular targets for “prostaglandin-associated enteropathy”.

## Materials and Methods

### Ethics statement

Written informed consent for genetic studies was obtained from each individual. The study was approved by the institutional review board at each collecting site in accordance with the Declaration of Helsinki Principles.

### Study participants

We obtained blood samples and family pedigrees from 17 Japanese patients with CNSU and eight unaffected family members in 15 families. The diagnosis of CNSU was based on the published clinical criteria and clinical courses (S3 Table) [8,29]. Genomic DNA samples from 747 participants in our previous genome-wide association study for ulcerative colitis [9] and 603 patients with CD [10,30] were used after excluding subjects who recalled their consent.

### Genetic analysis

DNA was extracted from peripheral blood using standard methods. Whole-exome sequencing in five affected individuals (A-V–2, B-IV–3, C-IV–3, D-II–4, and D-II–5) and one unaffected individual (A-V–3) was performed to identify candidate genetic variants (Fig 1). Genomic DNA was enriched using a TruSeq Exome Enrichment Kit (Illumina, San Diego, CA, USA) according to the manufacturer’s instructions, and paired-end sequencing was carried out with an Illumina HiSeq 2000 instrument. Reads were aligned to the human genome reference sequence (hg19 NCBI build 37.1) and decoy sequences using BWA software [31]. Duplicate reads were removed with the Picard program (http://picard.sourceforge.net/). Recalibration and realignment of the data were accomplished with Genome Analysis Toolkit (GATK) [32,33]. Single nucleotide variants and small insertions and deletions (indels) were identified by GATK Unified Genotyper. The effect of each missense mutation was predicted using SIFT (http://sift.jcvi.org/) [34], PolyPhen–2 (http://genetics.bwh.harvard.edu/pph2/) [35], and PROVEAN (http://provean.jcvi.org/) [36].

To compensate for bias in our analysis, such as the possibility of ethnic-specific variants, we genotyped the four candidate variants identified by exome sequencing in 747 unaffected Japanese subjects by Sanger sequencing and restriction fragment length polymorphism analysis (S4 Table). For further validation, Sanger sequencing of all exons of the *SLCO2A1* gene in other CNSU patients was performed using standard protocols. Finally, we genotyped the six identified mutation sites in the *SLCO2A1* gene in clinically diagnosed CD patients, because CNSU can be misdiagnosed as CD.

### Urinary prostaglandin measurement

Urine samples were collected from 15 CNSU patients and 13 unaffected individuals. The PGE-M levels were measured in duplicate using competitive enzyme-linked immunosorbent assays (Cayman Chemical, Ann Arbor, MI, USA).

### RNA analysis

We analyzed the exon 7 and exon 10 boundary mutations using RT-PCR to examine the effects of the splice-site mutations on *SLCO2A1* transcription. Total RNA was extracted from biopsy specimens of the small intestine and peripheral blood mononuclear cells using a NucleoSpin RNA Kit (Macherey-Nagel, Düren, Germany) or PAXgene Blood RNA Kit (Qiagen, Hilden, Germany). cDNA was synthesized using a PrimeScript First Strand cDNA Synthesis Kit (TaKaRa, Otsu, Japan). The PCR products obtained from the cDNAs were sequenced (S5 Table).

### Construction of expression vectors for the *SLCO2A1* gene

A full-length cDNA (NM_005630) expression vector and C-terminally GFP-tagged cDNA expression vector were purchased from OriGene Technologies (Rockville, MD, USA). To construct vectors carrying a mutated cDNA, a KOD -Plus- Mutagenesis Kit (Toyobo, Osaka, Japan) was used according to the manufacturer’s instructions. The expression vectors were amplified by inverse PCR with specific primer sets (S6 Table). The PCR products were self-ligated, and transformed into *Escherichia coli* chemically competent DH5α cells. To correct a frameshift in the downstream of exon 7, a C-terminally GFP-tagged cDNA expression vector with deletion of exon 7 was amplified again.

### Tracer PGE2 uptake analysis

On the day before transfection, HEK293 cells were trypsinized, counted, and plated onto 12-well plates at a density of 4×10^5^ cells/well. The cells were transfected by adding a premixed solution containing 0.4 μg of expression vectors and 2 μl of ScreenFectA (Wako, Osaka, Japan). After 24 hours of incubation, the medium was exchanged twice with warmed Waymouth’s medium (Life Technologies, Carlsbad, CA, USA), and the cells were incubated for 30 minutes at 37°C in uptake medium containing [5,6,8,11,12,14,15-^3^H(N)]-PGE2 (PerkinElmer, Waltham, MA, USA) at 0.6 nM. The cells were washed four times with cold Waymouth’s medium, and lysed with 200 μl of RIPA Buffer (Thermo Fisher Scientific, Hemel Hempstead, UK) containing a protease inhibitor (Roche, Basel, Switzerland). The total protein concentration was quantified using a BCA Protein Assay Kit (Thermo Fisher Scientific). Next, 150 μl of cell lysate was mixed with 5 ml of MicroScint–20 (PerkinElmer), and scintillation counting was performed in a Tri-Carb 3100TR liquid scintillation spectrometer (PerkinElmer).

### Immunohistochemistry and immunofluorescence staining

Formalin-fixed paraffin-embedded tissues were sectioned at 3-μm thickness. After antigen unmasking in 10 mM sodium citrate buffer (pH 6) for 15 minutes at 121°C, the sections were blocked with Protein Block Serum-Free (Dako, Glostrup, Denmark) for 30 minutes at room temperature. The sections were then incubated with a diluted anti-SLCO2A1 antibody (HPA013742; Sigma-Aldrich, St. Louis, MO, USA; antigen sequence: PSTSSSIHPQSPACRRDCSCPDSIFHPVCGDNGIEYLSPCHAGCSNINMSSATSKQLIYLNCSCVTGGSASAKTGSCPVPCAH) overnight at 4°C, followed by MAX-PO (MULTI) (Nichirei, Tokyo, Japan) for 30 minutes at room temperature. DAB solution (Nichirei) was applied for color development. After the immunocytochemistry, the sections were counterstained with Mayer’s hematoxylin solution (Nichirei). For immunofluorescence, the sections were incubated with a primary antibody mixture of the anti-SLCO2A1 antibody (HPA013742) and an anti-VE-cadherin antibody (LS-B3780; LifeSpan BioSciences, Seattle, WA, USA) overnight at 4°C, followed by a secondary antibody mixture of Alexa Fluor 568-conjugated goat anti-rabbit IgG (H&L) antibody and Alexa Fluor 488-conjugated goat anti-mouse IgG (H&L) antibody (Life Technologies) for 30 minutes at room temperature. The stained sections were analyzed using an ECLIPSE TE2000-U (Nikon, Tokyo, Japan).

For observation of HEK293 cells expressing GFP-fusion proteins, cells were fixed with 4% paraformaldehyde phosphate buffer solution (Wako) for 20 minutes, and then permeabilized with 0.1% Triton X–100 (Sigma-Aldrich) in D-PBS(-) solution (Wako) for 20 minutes. Nuclei were stained with 16.2 μM Hoechst 33342 (Life Technologies) in D-PBS(-) solution for 5 minutes. GFP and nuclei were visualized using a 40× objective on an LSM710 Laser Scanning Confocal Microscope (Carl Zeiss, Oberkochen, Germany).

### Statistical analysis

The chi-square test and Fisher’s exact test, where appropriate, were used to analyze categorical data. Student’s *t*-test was used to compare quantitative data between two groups. Dunnett’s method was used for multiple comparisons with a control group. The analyses were performed using JMP Pro statistical package 11.2.0 (SAS Institute, Cary, NC, USA). Values of *p* < 0.05 were regarded as statistically significant.

## Supporting Information

S1 Table Summary of whole-exome sequencing data. (PDF)

S2 Table Clinical manifestations of primary hypertrophic osteoarthropathy in patients with chronic nonspecific multiple ulcers of the small intestine. (PDF)

S3 Table Clinical criteria for chronic nonspecific multiple ulcers of the small intestine. (PDF)

S4 Table Primers for mutation analysis of the *SLCO2A1* gene. (PDF)

S5 Table Primers for mRNA analysis. (PDF)

S6 Table Primers for construction of expression vectors for mutant *SLCO2A1* genes. (PDF)

S1 Fig Concentrations of urinary prostaglandin E metabolite (PGE-M) in affected and unaffected individuals. The urinary levels of PGE-M are significantly elevated in CNSU patients (*n* = 15) compared with the levels in unaffected individuals (*n* = 13) (mean ± SEM: 116.1 ± 15.6 vs 35.9 ± 4.6 ng/mmol creatinine). *p < 0.001, by a two-tailed Student’s *t*-test.
(TIF)

S2 Fig Sequencing of RT-PCR products of mRNA transcripts. Patient A-V–2 has a homozygous c.1461+1G>C mutation that leads to a 23-bp frameshift insertion into intron 10, resulting in a premature stop codon (p.I488Lfs*11).
(TIF)
