# Supplementary material for: A Hereditary Enteropathy Caused by Mutations in the SLCO2A1 Gene, Encoding a Prostaglandin Transporter
Source: PLoS Genet. 2015 Nov 5;11(11):e1005581. doi: 10.1371/journal.pgen.1005581 (PMC4634957; doi:10.1371/journal.pgen.1005581)
Supplement: S1 Table — (PDF) [file pgen.1005581.s001.pdf]

S1 Table. Summary of Whole-exome Sequencing Data

Subjects	A-V-2	B-IV-3	C-IV-3	D-II-4	D-II-5	A-V-3 (unaffected
Total number of sequenced reads passing QC (×10^6)	103.5	111.0	93.1	117.6	97.9	111.6
Total number of mapped reads (×10^6)	103.1	110.4	92.4	117.2	97.5	110.8
Mean target coverage	66.7	71.2	55.8	82.8	65.1	71.8
% targets with 10× coverage	91.7	93.5	92.7	92.7	91.7	92.9
Number of identified variants						
SNV/indel	107,406	112,602	105,357	110,880	107,840	104,545
NS/SS	9,436	9,477	9,095	9,544	9,285	8,937
Number of candidate genes						
NS/SS (homozygous/heterozygous)	2,731/3,227	2,797/3,253	2,775/3,023	2,727/3,367	2,659/3,343	2,754/2,921
Not in dbSNP135 (homozygous/heterozygous)	**27**/286	**25**/265	**29**/257	18/294	16/265	22/159

SNV, single nucleotide variant; indel, insertion/deletion; NS, non-synonymous mutation; SS, splice-site mutation.
