# Supplementary material for: A Hereditary Enteropathy Caused by Mutations in the SLCO2A1 Gene, Encoding a Prostaglandin Transporter
Source: PLoS Genet. 2015 Nov 5;11(11):e1005581. doi: 10.1371/journal.pgen.1005581 (PMC4634957; doi:10.1371/journal.pgen.1005581)
Supplement: S2 Table — (PDF) [file pgen.1005581.s002.pdf]

S2 Table. Clinical Manifestations of Primary Hypertrophic Osteoarthropathy in Patients with Chronic Nonspecific Multiple Ulcers of the Small Intestine

Patients	1<br>A-V-2	2<br>B-IV-3	3<br>C-IV-3	4<br>D-II-4	5<br>D-II-5	6	7	8	9	10	11	12	13	14	15	16	17	18
Sex	F	F	F	F	F	F	F	F	F	F	M	M	F	F	F	M	M	F
Current age (yr)	57	75	58	61	59	51	42	52	72	59	42	63	56	40	18	70	28	75
Skeletal manifestations																		
Digital clubbing	+	-	-	-	-	-	-	-	+	-	-	+	-	-	-	+	+	+
Periostosis	-	-	-	NA	-	NA	NA	-	+	-	-	+	+	-	NA	+	+	+
Acro-osteolysis	-	-	-	NA	-	NA	NA	-	-	-	-	-	-	-	NA	-	-	-
Arthralgia of large joints	-	-	-	-	+	-	-	+	-	-	-	-	-	+	-	-	-	+
Knee-joint effusions	-	-	-	-	-	-	-	-	-	-	-	-	-	-	-	-	-	+
Skin manifestations																		
Hyperhidrosis	-	-	-	-	+	-	-	-	-	-	-	-	-	+	-	-	-	-
Pachydermia	-	-	-	-	-	-	-	-	-	-	-	+	-	-	-	+	+	-
Seborrhea	-	-	-	-	-	-	-	-	-	-	-	+	-	-	-	-	-	-
Acne	-	-	-	+	+	-	-	-	-	-	-	-	-	+	-	-	-	-
Flushing	-	-	-	-	+	-	-	-	-	-	-	+	-	-	-	-	+	-
Developmental manifestations																		
Patent ductus arteriosus	-	-	-	-	-	-	-	-	-	-	-	-	-	-	-	-	-	-
Delayed cranial suture closure	-	-	-	-	-	-	-	-	-	-	-	-	-	-	-	-	-	-

NA, not available.
