# Supplementary material for: A Hereditary Enteropathy Caused by Mutations in the SLCO2A1 Gene, Encoding a Prostaglandin Transporter
Source: PLoS Genet. 2015 Nov 5;11(11):e1005581. doi: 10.1371/journal.pgen.1005581 (PMC4634957; doi:10.1371/journal.pgen.1005581)
Supplement: S3 Table — (PDF) [file pgen.1005581.s003.pdf]

S3 Table. Clinical Criteria of Chronic Nonspecific Multiple Ulcers of the Small Intestine

1	Persistent and occult blood loss from the GI tract except during bowel rest or postoperative period.	
2	Confirmation of characteristic small intestinal lesions by macroscopy, radiography, or enteroscopy.	
	i	Circular or oblique in alignment.
	ii	Sharply demarcated from surrounding normal mucosa.
	iii	Geographic or linear in shape.
	iv	Multiplicity in number with < 4-cm distance from each other.
	v	Ulcers not reaching proper muscular layer.
	vi	Scarred ulcers presumed to be the healing stage of those characterized by i-v* in cases treated by bowel rest.

*Depicted as symmetric and eccentric rigidity under small-bowel radiography, and concentric or nonconcentric stricture under enteroscopy. GI, gastrointestinal.
