# Supplementary material for: A Hereditary Enteropathy Caused by Mutations in the SLCO2A1 Gene, Encoding a Prostaglandin Transporter
Source: PLoS Genet. 2015 Nov 5;11(11):e1005581. doi: 10.1371/journal.pgen.1005581 (PMC4634957; doi:10.1371/journal.pgen.1005581)
Supplement: S4 Table — (PDF) [file pgen.1005581.s004.pdf]

S4 Table. Primers for Mutation Analysis of *SLCO2A1* Gene

Amplicon	Forward (5′–3′)	Reverse (5′–3′)	Size (bp)	RFLP*
Exon 1	GCCTGGAGTTGTCCGAGTAAG	TTTGACACCCGAGGAAAAGAG	620	
Exon 2	ACAGAATTTGAAGGCGGACAG	TGATGACGTTTCATCCTGGAG	655	
Exon 3	AATGCTTTGATCCTCCTCCTG	CAGAGGGAAAACCAAAATTGC	676	
Exon 4	GCTGTTGGTTTAGGGTCCATC	CCCACATTCCACCTCTCTTTC	786	
Exon 5	AAGGGCAGAAAGTGAGTTTGC	GCAGGTCTCTTTGGAAGTTGG	570	
Exon 6	AATTTCAGCCACCCTCAACAC	CAATAGCTGGGAGAGGGAATG	742	
Exon 7-8	GATTGCCAACCAGGAAAACTG	AGGTGCTGTTTGCTTCAGGAG	778	*HpyCH4IV*
Exon 9	AGTTGCCACGTGAGAGATCAG	GTGTAGGCAAGGCAATCCTG	860	
Exon 10	TGCTTTGACTGGTTTCTGCTC	TGGTTGTCTTGACGCCTACTG	747	*HapII, BsrI*
Exon 11	GCAAAAGAACCTTGCACATTG	GCCTCCCTCTGCAATAAACAC	710	
Exon 12	AAACCGTCCACATGGATTTTG	CAGCATCCTTCTCTCCACTCC	643	
Exon 13	GTGGCCCTTCATGTTCTCTTC	GCCCGTGTATCTCCACTCTG	564	
Exon 14	CAAATGAGGACTGGGGTTTTC	GAAGACCACAAAAGGGACAGG	617	

**HpyCH4IV*, *HapII*, and *BsrI* were used for genotyping of c.940+1G>A, c.1372G>T, and c.1461+1G>C mutations, respectively. RFLP, restriction fragment length polymorphism.
