# Supplementary material for: A Hereditary Enteropathy Caused by Mutations in the SLCO2A1 Gene, Encoding a Prostaglandin Transporter
Source: PLoS Genet. 2015 Nov 5;11(11):e1005581. doi: 10.1371/journal.pgen.1005581 (PMC4634957; doi:10.1371/journal.pgen.1005581)
Supplement: S5 Table — (PDF) [file pgen.1005581.s005.pdf]

S5 Table. Primers for mRNA Analysis

Amplicon	Forward (5′–3′)	Reverse (5′–3′)	Size (bp)
Exon 6-9	AGGCCTGCTCATTTCTTCAG	ACAAAGCGCTTCATGAGGAT	396
Exon 8-13	GCACCTTCTCCTCCGTCATTG	CGTTGTCATAGTAGGCGCAGG	792
