# Supplementary material for: A Hereditary Enteropathy Caused by Mutations in the SLCO2A1 Gene, Encoding a Prostaglandin Transporter
Source: PLoS Genet. 2015 Nov 5;11(11):e1005581. doi: 10.1371/journal.pgen.1005581 (PMC4634957; doi:10.1371/journal.pgen.1005581)
Supplement: S6 Table — (PDF) [file pgen.1005581.s006.pdf]

S6 Table. Primers for Construction of Expression Vectors for Mutant *SLCO2A1* Genes

Mutation	Forward (5′–3′)	Reverse (5′–3′)
c.940+1G>A	GGTTTCCATGCATCTTTCTGAGGC	CTTTGCTCCTATGGGCATTGCTC
c.940+1G>A (for GFP-SLCO2A1)	ACGCGTACGCGGCCGCTCGAG	GAAAGATGCATGGAAACCCTTTGC
p.Gly222Arg	AGGTACCTGCTGGGCTCTGTCATGCTGC	GAAAGCCGGTCCAAATACAGAGATGGC
p.Arg603X	TGAGACAGGTACCTGGGCCTGCAGATGGG	GAGAGCATCGTTGTCATAGTAGGCGCAGG
p.Glu141X	TAGCTCTGCCAGAAGCATTGGCAGGACC	GGCCTGCAAGCGGCTGTTGTTCCCAGTGC
p.Gly183Arg	AGGACAGTGCCTATTCAGCCATTTGGG	GATGCCAGCCAGCAGCTGGGCAACCACC
p.Val458Phe	ACCCGTTCTGTGGAGACAATGGAATCGAGTACC	GGAAGATAGAATCTGGGCACGAGCAGTCCC
