# Supplementary figures and images for: A Hereditary Enteropathy Caused by Mutations in the SLCO2A1 Gene, Encoding a Prostaglandin Transporter
Source: PLoS Genet. 2015 Nov 5;11(11):e1005581. doi: 10.1371/journal.pgen.1005581 (PMC4634957; doi:10.1371/journal.pgen.1005581)

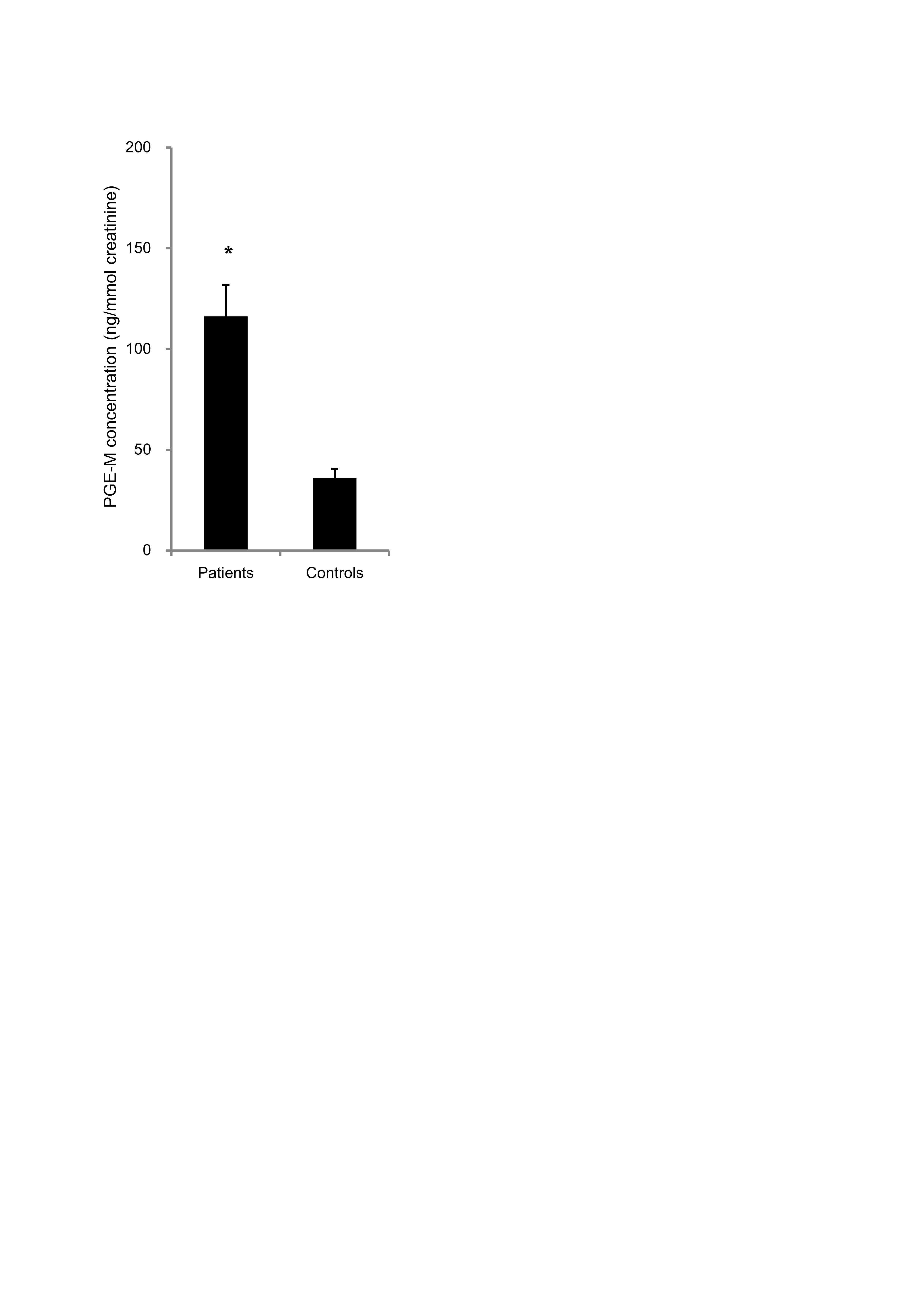

Supplement: S1 Fig — The urinary levels of PGE-M are significantly elevated in CNSU patients (n = 15) compared with the levels in unaffected individuals (n = 13) (mean ± SEM: 116.1 ± 15.6 vs 35.9 ± 4.6 ng/mmol creatinine). *p < 0.001, by a two-tailed Student’s t-test. (TIF) [file pgen.1005581.s007.TIF]

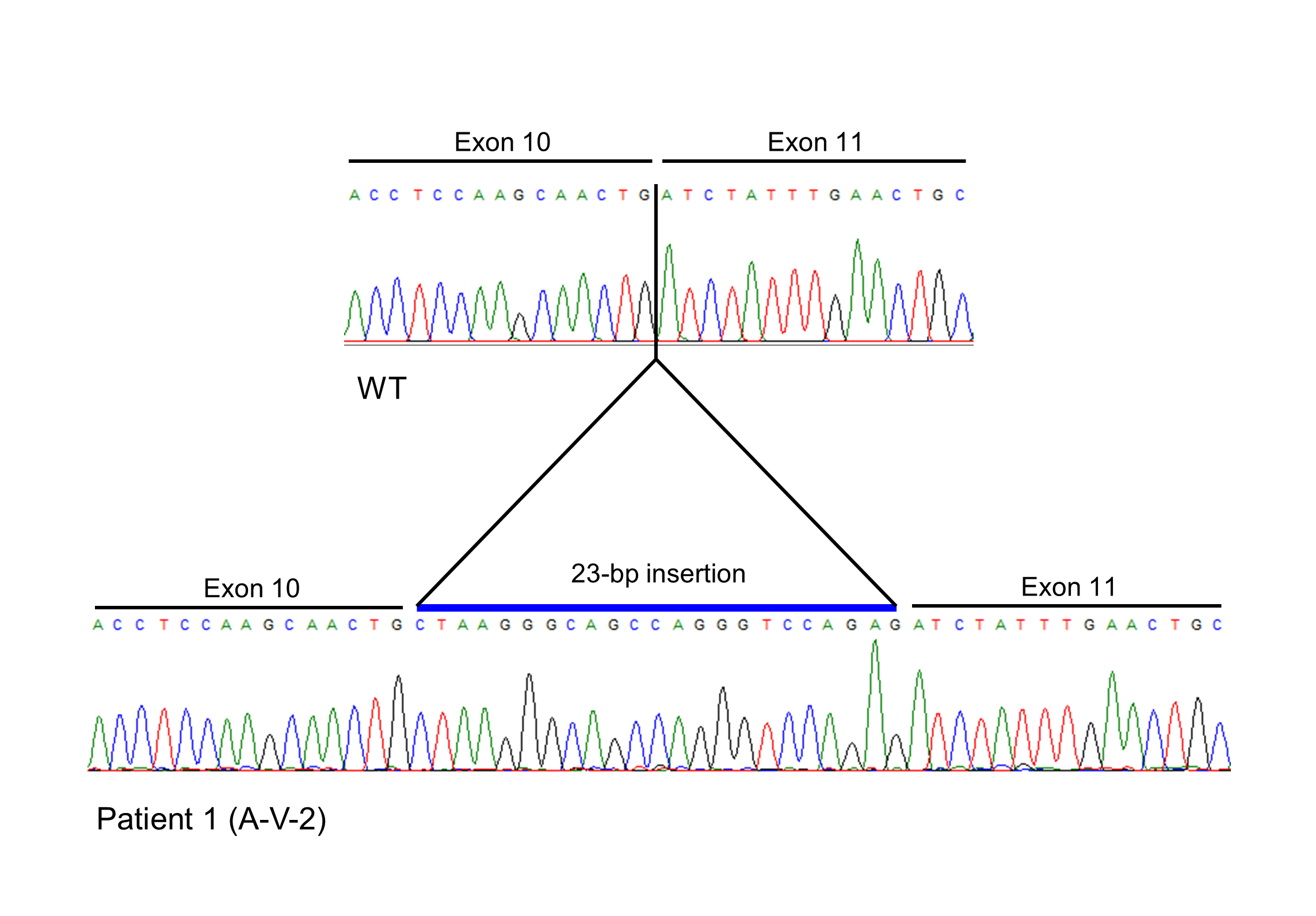

Supplement: S2 Fig — Patient A-V–2 has a homozygous c.1461+1G>C mutation that leads to a 23-bp frameshift insertion into intron 10, resulting in a premature stop codon (p.I488Lfs*11). (TIF) [file pgen.1005581.s008.TIF]
